# Technology-facilitated sexual violence among Italian youths: validation of the technology-facilitated sexual violence victimization scale

**DOI:** 10.3389/fpsyt.2024.1449183

**Published:** 2024-11-01

**Authors:** Laura Orsolini, İmran Gökçen Yılmaz-Karaman, Kerim Selvi, Salvatore Reina, Giulio Longo, Umberto Volpe

**Affiliations:** ^1^ Unit of Clinical Psychiatry, Department of Clinical Neurosciences/DIMSC, Polytechnic University of Marche, Ancona, Italy; ^2^ Department of Psychiatry, Faculty of Medicine, Eskişehir Osmangazi University, Eskişehir, Türkiye; ^3^ Department of Life, Health and Environmental Sciences, University of L'Aquila, L'Aquila, Italy; ^4^ Department of Psychology, Eskişehir Osmangazi University, Eskişehir, Türkiye

**Keywords:** Sexual and gender minority (SGM), sexual violence, technology-facilitated sexual violence, TFSV, victimization

## Abstract

**Introduction:**

Technology-facilitated sexual violence (TFSV), i.e. the use of digital communication technologies for facilitating sexual violence and harassment, represents a concern due to its exponential growth, particularly among youths. Few studies investigated TFSV, mainly due to the lack of a validated assessment tool, such as the TFSV-Victimization scale (TFSV-VS). Our study aimed to investigate the TFSV phenomenon in a sample of Italian young adults (aged 18-24), through the validation of the Italian translated version of TFSV-VS.

**Methods:**

The study consisted of two phases: 1) validation of the Italian version of TFSV-VS; b) evaluation of TFSV lifetime and during the last 12 months among Italian youths.

**Results:**

In our sample, 78.6% and 56.1% of subjects reported a lifetime and 12-months TFSV victimization, respectively. Digital sexual (70.4% and 46.6%), gender and/or sexuality-based harassment (43% and 29.6%) are those most represented. A gendered and sexuality-based pattern in lifetime TFSV was observed, mainly observed among females (p=0.005) and sexual/gender minority (SGM)(p=0.001). Being female (*p*<0.001) and perceiving low social support (*p* = 0.030) were associated with higher subjective distress related to traumatic TFSV experiences. Being female (*p*<0.001), younger (*p*=0.006) and perceiving low social support (*p*=0.030) were associated with the highest psychological distress due to TFSV.

**Conclusions:**

Italian TFSV-VS shows good psychometric properties. Our findings observed a gendered trend of TFSV, by suggesting TFSV as a phenomenon influenced by SGM belonging.

## 1 Introduction

With advances in technology and the spread of social media, social interactions between individuals now take place both in-person and online (1). Particularly during the COVID-19 pandemic, online contacts have been and are becoming increasingly important in daily relationship life, especially among young people, due to the limitations in travel to crowded environments for preventing disease transmission (2). In fact, most online interactions were mainly created and grown up during the COVID-19 pandemic, and currently they are still maintained and implemented in our daily life (3). Indeed, also virtual/remote interpersonal relationships may determine significant implications on each individual’s daily life, both negatively and positively (4). In the context of online interactions, an individual could potentially use the virtual tool to meet up other people with similar difficulties and/or issues and share their own experiences/feelings and emotions in a more or less functional way and, hence, potentially provide and receive good social support (5). However, at the same time, just like any real-life encounters, these virtual interpersonal interactions can put any subject in the position of potentially experiencing and/or being exposed to virtually mediated interpersonal violence, cyberbullying, and so forth (6).

Technology-facilitated violence represents a form of digital interpersonal violence that is committed and amplified through the use of Information and Communications, technologies and digital spaces (7). Technology-facilitated violence may take many forms, including sextortion (i.e., blackmail by threatening to publish sexual information, photos or videos), image-based abuse (i.e., sharing intimate photos without consent), doxxing (i.e., publishing private personal information), cyberbullying (i.e., bullying with the use of digital technologies, it can take place on social media chats, messaging platforms, gaming platforms and more), online sexual harassment (i.e., a form of sexual harassment that primarily occurs over the Internet, typically through e-mail, an Internet forum, or online messaging programs), cyberstalking (i.e., using technology to harass and intimidate victims), online grooming for sexual assault (i.e., building trust and making connections with someone to get him to do something sexual), hate speech, online impersonation (i.e., using an identity other than their own for malicious purposes), hacking (i.e., stealing personal information or banking accounts), and so forth (8, 9). Moreover, as technology-facilitated violence is more frequently expressed through emotional abuse and sexual harassment, perpetrators may be more prone to exploit social virtual spaces or using online video games (particularly chats associated with videogames) to select and then meet their victims in the real life to inflict physical and sexual violence (10).

Indeed, since the beginning of the COVID-19 pandemic, there has been a significant increase in the number of calls made by women to emergency/support numbers to notify, or even denounce, episodes of technology-facilitated physical, sexual, psychological violence or intimate partner violence (IPV) (11, 12). However, currently, our knowledge regarding this type of technology-facilitated sexual violence is scanty, primarily based on studies on children and adolescents (13–15). While data on technology-facilitated violence prevalence among young adults is limited, particularly in the Italian context (16, 17).

Overall, only one assessment tool is currently available for measuring technology-facilitated violence, i.e. the technology-facilitated sexual violence-victimization scale (TFSV-VS) (6), a 21-item scale developed in accordance with prior conceptual research identifying multiple dimensions of TFSV-VS including digital sexual harassment, image-based sexual abuse, sexual aggression and/or coercion, and, gender and/or sexuality-based harassment (including virtual sexual violence) (6). However, the instrument is only available in its English and Spanish version. No validated Italian translated tools are currently available for assessing TFSV in the Italian sample. The TFSV-VS developed by Powell and Henry (6) could effectively represent a valid tool to measure the phenomenon of technology-facilitated violence, also in the Italian context. Therefore, the goal of the present study was to validate the Italian version of the TFSV-VS proposed by Powell and Henry (6) as well as investigating the TFSV phenomenon in a sample of Italian young adults (aged 18-24).

## 2 Materials and methods

### 2.1 Translation of the TFSV Victimization scale, study design and recruitment strategies

A written formal authorization was firstly obtained from Anastasia Powel, one of the researchers who developed the original English instrument TFSV-VS (18), to translate and validate the questionnaire into Italian language. The translation process was carried out by three researchers fluent in Italian and English. Then, the best translations based on their semantic similarity with the original scale items were selected, revised, and created from scratch. Also, the evaluation of a cultural adaptation of the scale was also achieved and established after a discussion within the research team, until an unanimous consensus about the Italian version was achieved, when the level of agreement among team members was greater than 50%. These selected translated items were then back-translated into English by a bilingual native English-speaking researcher to compare the newly translated version with the original questionnaire. The final version of all items were decided by examining the original and translated items. The translated version is available upon request to the corresponding author. The study was conducted in accordance with the ethical principles outlined in the Declaration of Helsinki and according to the guidelines for Good Clinical Practice (GCP). The study was approved by the local Institutional Review Board of the Department of Clinical and Experimental Medicine of the Polytechnic University of Marche (protocol code ACPS-D-21-00347, 28 September 2021).

### 2.2 Validation of the TFSV Victimization scale and recruitment strategy

The Italian translated version of the TFSV-VS questionnaire was uploaded to Qualtrics platform for online surveys (www.qualtrics.com) and the study link was announced to potential participants. The study was carried out during the timeframe January, 2022-February, 2023. The sample consisted of a cohort of Italian university students randomly selected. The inclusion criteria were: a) 18-25-years-old; b) agreement to participate in the study according to the informed consent form, which all participants signed before participating in the study. Once they clicked on the study link, participants were informed about the purpose of the study and their answers were collected anonymously. Their participation was voluntary and they could leave the study at any time without giving any reason. After obtaining their online informed consent, the participants filled out the questionnaires, which took about 10-15 minutes. The questionnaire was administered initially at three different times: the first (T0) was overseen by researcher A; the second (T1) occurred 15 min after the end of T0 and was overseen by researcher B; and the third (T2), 15 days later, when the instrument was completed after being sent by researcher A.

### 2.3 Measures

#### 2.3.1 Demographic information form

This form was used to collect participants’ socio-demographic information about age, gender, sexual orientation, perceived income level, and usage of social media/Internet and dating/dating sites or apps.

#### 2.3.2 Technology facilitated sexual violence-victimization scale

The TFSV-VS was developed by Powell and Henry (6) to measure the subjective experience of TFSV victimization. It consists of 21 items and four sub-factors, namely digital sexual harassment/intrusion (e.g., “Posted personal details online saying you are available to have sex”), image-based sexual abuse (e.g., “Nude or semi-nude image threat to post online/send onto others”), sexual aggression/coercion (e.g., “Unwanted sexual experience with someone met online”), and gender/sexuality-based harassment (e.g., Gender-based offensive and/or degrading messages, comments, or other content”). Participants are asked to rate each item as never in their life, once, and more than once in the last 12 months. In the original study, the internal consistency reliability of the whole scale was 0.93. The Italian translation of TFSV-VS was performed in the current study, and the response options were modified to obtain TFSV victimization experiences that occurred before the last twelve months. In this version, the respondents were requested to rate each item on a 3-point Likert-type scale (*0* = never, *1* = once, and *2* = more than once) for their lifetime and the last 12 months. Thus, higher scores on the TFSV-VS indicate higher subjective experiences of TFSV victimization.

#### 2.3.3 The multidimensional scale of perceived social support

The MSPSS is a self-report instrument to assess participants’ perceived social support from three sources, i.e., family, friends, and significant others. It was developed by Zimet et al. (19) and translated into Italian by Prezza and Principato (20) following the backward-forward translation method, and later validated by Di Fabio and Busoni, (21). It consists of 12 items (e.g., “There is a special person who is around when I am in need.”) rated on a 7-point Likert-type scale ranging from 1 (*Very strongly disagree*) to 7 (*Very strongly agree*). Thus, higher scores on the MSPSS reflect higher perceived social support. The internal consistency reliability coefficients of the original and the Italian version of MSPSS were 0.88 and 0.91, respectively. In the present study, the composite score of the MSPSS was used, and Cronbach’s alpha coefficient was 0.90.

#### 2.3.4 Kessler psychological distress scale

It is a self-report scale developed by Kessler et al. (22) to measure psychological distress (i.e., depression and anxiety symptoms). It consists of 10 items (e.g., “During the past 30 days, about how often did you feel hopeless?”) rated on a 5-point Likert-type scale ranging from 1 (*None of the time*) to 5 (*All of the time*). Hence, higher scores on the K10 indicate higher psychological distress. The Italian adaptation of K10 was done by Carrà et al. (23), and the internal consistency reliability of this version was 0.90. In the current study, the Italian version of K10 was used to assess the participants’ psychological distress, and Cronbach’s alpha coefficient was 0.92.

#### 2.3.5 Impact of events scale-revised

The IES-R, a revised version of the IES (24), is a self-report that measures subjective distress related to traumatic events. It consists of 22 items (e.g., “I had trouble staying asleep.”) rated on a 5-point Likert-type scale ranging from 0 (*Not at all*) to 4 (*Extremely*). Hence, higher scores on the IES-R indicate higher trauma-related subjective distress. The IES-R includes three sub-factors, namely avoidance, intrusion, and hyperarousal. The IES-R was translated into Italian language by Craparo et al. (25), and the internal consistency reliabilities of the Italian version were 0.72 for avoidance, 0.78 for intrusion, and 0.83 for hyperarousal. In the current study, the composite score of the IES-R was used, and Cronbach’s alpha coefficient was 0.98.

### 2.4 Statistical analysis

First, the validity and reliability of Italian TFSV-VS were examined. Regarding the validity of Italian TFSV-VS, Pearson zero-order correlation analysis was performed to see the correlations of TFSV-VS with the IES-R and K10 scores and the average internet and social media usage time per day. For divergent validity, it was expected that TFSV-VS would not significantly correlate with theoretically irrelevant variables, i.e., the average internet and social media usage time per day. To convergent validity, it was expected that TFSV-VS would significantly and positively correlate with theoretically relevant constructs, i.e., the IES-R and K10 scores. Also, the reliability of Italian TFSV-VS was examined via internal consistency reliability. Then, the frequency of participants’ TFSV experiences in the last 12 months and their lifetime was examined. Also, several chi-square tests of independence were performed to test whether TFSV-VS experiences in the last 12 months and the lifetime change depend on the gender and sexual orientations of the participants. If at least one chi-square assumption was violated, the results of Fisher’s exact tests were reported instead of the results of chi-square independence tests. To test the predictive association of lifetime TFSV with psychological distress (i.e., the score of K10) and trauma-related stress (i.e., the score of IES-R), two regression analyses with the enter method were performed. In each regression analysis, as control variables, demographic variables (i.e., gender, age, sexual orientation, and perceived income level) were entered into the equation in the first step, and the score of MSPSS was entered into the equation in the second step. In addition to the regression analyses, two moderation analyses using Hayes’s Process Macro (26) were performed, considering that the relationship between lifetime TFSV with psychological distress and trauma-related stress might change depending on the levels of social support. As lifetime TFSV covers technology-assisted victimization experiences in the last 12 months, the composite score of lifetime TFSV was used as the predictor in the regression and moderation analyses. For all analyses, the level of statistical significance was set at p<0.05, two-tailed. All statistical analyses were performed using the software Statistical Package for Social Science (SPSS) version 27.0 for MacOS (IBM SPSS Statistics, Chicago, IL, United States).

## 3 Results

### 3.1 Sample characteristics

Although 358 university students participated in the study in the first phase, 51 participants were excluded based on the inclusion criterion of the study (i.e., being between the ages of 18 and 25). Of the rest, 83 were excluded because they had not filled out several study scales including the TFSV-VS. However, those participants who did not complete only the IES-R were kept. Thus, we ended up with a final sample of 223 participants. Of the sample, 152 subjects were females (68.2%), while 71 were males (31.8%). Most of them (*n* = 185, 83.0%) reported their sexual orientation as heterosexual, whereas the rest reported it as ‘other’. The mean age of the participants was 21.7 (*SD* = 2.1), without any sex-based differences (*p* = 0.077). Most of them (*n* = 158, 70.9%) reported their perceived income level as moderate, and the rest reported as either low (*n* = 42, 18.8%) or high (*n* = 23, 10.3%). Most participants reported to have had an affective relationship lasted more than one month (*n* = 181, 81.2%). Almost all participants (*n* = 217, 97.3%) reported having at least one social media account. Fifty-six participants (25.1%) reported using online dating/dating sites or apps. The average hours of their Internet usage per day was 4.9 (*SD* = 2.7, range = 1 – 16). Overall, our sample is mainly constituted by subjects without a previous psychiatric and/or psychological consultation (*n* = 124, 55.6%) and without previous and/or current psychopharmacological and/or psychological treatment (*n* = 191, 85.7%).

### 3.2 Psychometric properties of the Italian version of TFSV-VS

For the validity of Italian TFSV-VS, the correlations of TFSV-VS with the IES-R and K10 scores and the average internet and social media usage time per day were examined. Regarding convergent validity, as expected the TFSV-VS and its subscales were significantly and positively correlated with the scores of the IES-R and the K10 (except for a few nonsignificant correlations between TFSV-VS subscales and the score of K10). To divergent validity of the TFSV victimization scale, as expected, the average internet usage time per day was not significantly correlated with lifetime TFSV (*r* = 0.12, *p* = 0.123) and TFSV in the last 12 months (*r* = 0.08, *p* = 0.245). Consistently, the use of online dating/dating sites or apps was not significantly correlated with lifetime TFSV victimization (*r* = 0.02, *p* = 0.751) and TFSV in the last 12 months (*r* = -0.01, *p* = 0.898) (**Table 1**). The reliability of TFSV-VS was tested via the internal consistency reliability. The Cronbach’s alpha coefficients of lifetime TFSV victimization and TFSV victimization in the last 12 months were 0.75 and 0.76, respectively. However, Cronbach’s alpha coefficients of TFSV-VS subscales were between 0.52 and 0.69 for the lifetime experiences and between 0.61 and 0.64 for the experiences in the last 12 months (**Table 1**).

**Table 1:** Mean and standard deviation values of the study variables, bivariate correlations of study variables, and internal consistency coefficients of the scales.

Variables	M	SD	1	2	3	4	5	6	7	8	9	10	11	12	13	14	15	16	17
1. Age	21.71	2.08	–																
2. Perceived income level	1.91	.53	-.01	–															
3. Average time on the Internet per day (hour)	4.89	2.72	-.07	-.13	–														
4. Use of online dating/dating sites or apps (0 = no, 1 = yes)	.97	.16	-.02	.03	.00	–													
5. L-TFSV	4.57	4.48	.05	.05	.12	.02	**(.75)**												
6. L-DSH	2.76	2.73	.09	.06	.14*	.05	.88***	**(.66)**											
7. L-IBSA	.25	.77	-.02	-.00	.05	.02	.49***	.37***	**(.69)**										
8. L-SAC	.36	.88	.08	.01	.07	.01	.54***	.35***	.18**	**(.59)**									
9. L-GSBH	1.20	1.77	-.03	.01	.03	-.03	.69***	.36***	.15*	.24***	**(.52)**								
10. TFSV-12M	2.48	3.60	-.10	.03	.08	-.01	.76***	.62***	.35***	.48***	.57***	**(.76)**							
11. DSH-12M	1.40	2.08	-.08	.05	.08	-.02	.64***	.69***	.27***	.38***	.24***	.85***	**(.64)**						
12. IBSA-12M	.08	.43	-.10	-.05	.01	.03	.32***	.30***	.52***	.22**	.02	.52***	.48***	**(.62)**					
13. SAC-12M	.17	.67	-.04	-.02	.09	.04	.43***	.24***	.22**	.69***	.27***	.67***	.45***	.41***	**(.62)**				
14. GSBH-12M	.83	1.63	-.08	.03	.03	-.02	.60***	.31***	.20**	.23**	.84***	.71***	.29***	.11	.38***	**(.61)**			
15. MSPSS	65.30	13.06	.04	.06	-.01	.11	-.09	-.01	.03	-.11	-.17*	-.18**	-.09	-.06	-.18**	-.19**	**(.90)**		
16. K10	25.27	8.21	.14*	-.09	.06	.00	.24***	.22**	.09	.07	.19**	.18**	.17**	-.01	.06	.16*	-.35***	**(.92)**	
17. IES-R	12.29	19.67	.00	-.02	.08	.01	.71***	.64***	.43***	.54***	.40***	.47***	.38***	.31***	.36***	.35***	-.10	.21*	**(.98)**

*p <.05, **p <.01, ***p <.001.

L-TFSV, Total Lifetime TFSV victimization; L-DSH, Lifetime digital sexual harassment victimization; L-IBSA, Lifetime image-based sexual abuse victimization; L-SAC, Lifetime sexual aggression and/or coercion victimization;, L-GSBH, Lifetime gender and/or sexuality-based harassment victimization; TFSV-12M, TFSV victimization in the last 12 months; DSH-12M, Digital sexual harassment victimization in the last 12 months; IBSA-12M, Image-based sexual abuse victimization in the last 12 months; SAC-12M, Sexual aggression and/or coercion victimization the last 12 months; GSBH-12M, Gender and/or sexuality-based harassment victimization in the last 12 months; MSPSS, Multidimensional Scale of Perceived Social Support; K10, Kessler Psychological Distress Scale; IES-R, Impact of Event Scale – Revised.

Values shown in bold in parentheses are Cronbach’s alpha coefficients.

### 3.3 Prevalence of TFSV victimization

Of the participants, 78.9% (*n* = 176) experienced at least one TFSV victimization episode in their lifetime, whereas 21.1% (*n* = 47) have not experienced any TFSV victimization in their lifetime. For the facets of TFSV victimization in the lifetime, 70.4% (*n* = 157) reported at least one experience of digital sexual harassment, 14.8% (*n* = 33) reported at least one experience of image-based sexual abuse, 19.3% (*n* = 43) reported at least one experience of sexual aggression and/or coercion, and 43% (*n* = 96) reported at least one experience of gender and/or sexuality-based harassment. According to the results of chi-square tests of independence, the combined lifetime TFSV victimization differed by participants’ gender, *X^2^* (1, *N* = 223) = 8.02, *p* = 0.005. Females were more likely than males to be exposed to lifetime TFSV victimization. For the facets of lifetime TFSV victimization, a significant gender difference was found for digital sexual harassment [*X^2^* (1, *N* = 223) = 8.01, *p* = 0.005] and gender and/or sexuality-based harassment victimization [*X^2^* (1, *N* = 223) = 11.27, *p* = 0.001] but not for image-based sexual abuse [*X^2^* (1, *N* = 223) = 2.02, *p* = 0.156] and sexual aggression and/or coercion victimization [*X^2^* (1, *N* = 223) = 2.92, *p* = 0.087]. These findings indicated that females were more likely than males to be exposed to lifetime digital sexual harassment and gender and/or sexuality-based harassment victimization. The results of chi-square tests of independence examining if the lifetime TFSV victimization differs by participants’ sexual orientations showed a significant sexual orientation difference for the combined lifetime TFSV victimization [*X^2^* (1, *N* = 223) = 4.79, *p* = 0.029] and its facets of digital sexual harassment [*X^2^* (1, *N* = 223) = 4.19, *p* = 0.041] and gender and/or sexuality-based harassment victimization [*X^2^* (1, N = 223) = 7.56, *p* = 0.006] but not for image-based sexual abuse [*X^2^* (1, *N* = 223) = 0.10, *p* = 0.755] and sexual aggression and/or coercion victimization [*X^2^* (1, *N* = 223) = 0.57, *p* = 0.450]. Accordingly, the participants with sexual orientations other than heterosexual were more likely to be exposed to digital sexual harassment, gender and/or sexuality-based harassment victimization, and the combined lifetime TFSV victimization in their lifetime.

For TFSV victimization in the last 12 months, 56.1% of the participants (*n* = 125) experienced at least one TFSV victimization. For the facets of TFSV victimization, 46.6% (*n* = 104) reported at least one experience of digital sexual harassment, 3.6% (*n* = 8) reported at least one experience of image-based sexual abuse, 8.1% (*n* = 18) reported at least one experience of sexual aggression and/or coercion, and 29.6% (*n* = 66) reported at least one experience of gender and/or sexuality-based harassment in the last 12 months. The results of chi-square tests of independence showed that there was no gender difference for the combined TFSV victimization [*X^2^* (1, *N* = 223) = 1.93, *p* = 0.165], digital sexual harassment victimization [*X^2^* (1, *N* = 223) = .10, *p* = 0.749], sexual aggression and/or coercion victimization [X*^2^* (1, *N* = 223) = 1.43, *p* = 0.231] in the last 12 months. For image-based sexual abuse in the last 12 months, the Fisher’s exact test was conducted because one of the chi-square tests of independence assumptions (i.e., expected value of cells should be 5) was violated. According to the results of the Fisher’s exact test, image-based sexual abuse victimization in the last 12 months did not change based on gender (p = 0.269). Only significant gender difference was present for gender and/or sexuality-based harassment victimization in the last 12 months, *X^2^* (1, *N* = 223) = 14.31, *p* < 0.001. Accordingly, females were more likely than males to be exposed to gender and/or sexuality-based harassment victimization. Besides, chi-square tests of independence and Fisher exact test were performed to examine whether the TFSV victimization in the last 12 months differs by participants’ sexual orientations. The results revealed a significant sexual orientation difference only for gender and/or sexuality-based harassment victimization, *X^2^* (1, *N* = 223) = 9.15, *p* = 0.002. Accordingly, the participants with sexual orientations other than heterosexual were more likely to be exposed to gender and/or sexuality-based harassment victimization in the last 12 months. However, no significant difference was found for sexual aggression and/or coercion victimization (the Fisher’s exact test, *p* = .269), image-based sexual abuse [*X^2^* (1, N = 223) = 2.46, *p* = 0.117], digital sexual harassment [*X^2^* (1, *N* = 223) = 0.66, *p* = 0.416], and the combined TFSV victimization [*X^2^* (1, *N* = 223) = 2.84, *p* = 0.092].

### 3.4 Regression analyses

In the first regression analysis, the predictive association of lifetime TFSV victimization with subjective distress related to traumatic experiences (i.e., the scores of IES-R) were verified. According to the results, the control variables in the first step (i.e., gender, age, sexual orientation, and perceived income level) together explained a significant proportion of variance in the scores of IES-R, F (4, 150) = 4.77, R2 = 0.11, p = 0.001. However, of these variables, in the first step, only gender (coded as 1 = female, 2 = male) was a significant predictor of subjective distress related to traumatic experiences. More specifically, being female was associated with higher subjective distress related to traumatic experiences, β = -0.34, t (150) = -4.30, p < 0.001, ηp2 = -0.33. In the second step, perceived social support significantly explained an additional proportion of variance in the IES-R scores, ΔF (1, 149) = 4.80, ΔR2 = 0.03, p = 0.030. Accordingly, perceived social support negatively and significantly predicted the scores of IES-R, β = -0.17, t (149) = -2.19, p = 0.030, ηp2 = -0.18 (**Table 2**).

**Table 2:** Hierarchical linear regression model with IES-R total score (as dependent variable).

Predictors	B	SE	*β*	*t*	95%IC Lower Limit	95%IC Upper Limit	p-value	∆F	∆R^2^
Step 1							.001	4.77	.11
1. Gender (coded as 1 = female, 2 = male)	-13.82	3.22	-.34	-4.30	-20.18	-7.47	< .001		
2. Age (in years)	-.48	.75	-.05	-.65	-1.97	1.00	.520		
3. Sexual orientation (coded as 1 = heterosexual, 2 = other)	3.08	3.92	.06	.79	-4.67	10.82	.434		
4. Perceived income level	-.37	2.78	-.01	-.13	-5.86	5.13	.895		
Step 2							.030	4.80	.03
5. MSPSS	-.25	.11	-.17	-2.19	-.48	-.03	.030		
Step 3							< .001	113.85	.37
6. L-TFSV	2.76	.26	.68	10.67	2.52	3.28	< .001		

Note. SE, Standard Error; CI, Confidence Interval; MSPSS, Multidimensional Scale of Perceived Social Support; L-TFSV, Total Lifetime TFSV victimization; IES-R, Impact of Event Scale – Revised.

In the third step, lifetime TFSV victimization significantly explained an additional proportion of variance in the IES-R scores, ΔF (1, 148) = 113.85, ΔR2 = .37, p < 0.001. Lifetime TFSV victimization positively and significantly predicted the scores of IES-R, β = 0.68, t (148) = 10.67, p < 0.001, ηp2 = 0.66 (**Table 2**).

The second regression analysis was run to test the predictive association of lifetime TFSV victimization with psychological distress (i.e., the scores of K10). As in the first regression analysis, gender, age, sexual orientation, perceived income level, and social support were entered into the equation as control variables. The results showed that demographic variables explained a significant proportion of variance in the scores of K10 (psychological distress), F (4, 218) = 5.66, R2 = .09, p <.001. Of these variables, gender [β = -0.26, t (218) = -3.94, p < 0.001, ηp2 = -0.26] and age [β = -0.18, t (218) = -2.76, p = 0.006, ηp2 = -0.18] were negative and significant predictors of psychological distress, indicating that being female and younger is associated with higher psychological distress In the second step, perceived social support significantly explained an additional proportion of variance in the K10 scores, ΔF (1, 217) = 442.53, ΔR2 = 0.15, p < 0.001. Accordingly, perceived social support negatively and significantly predicted psychological distress, β = -0.39, t (217) = -6.52, p = 0.030, p < 0.001, ηp2 = -0.41. In the last step, lifetime TFSV victimization significantly explained an additional proportion of variance in the K10 scores, ΔF (1, 216) = 5.96, ΔR2 = 0.02, p = 0.015. Lifetime TFSV victimization positively and significantly predicted the scores of K10, *β* = 0.15, *t* (216) = 2.44, *p* = 0.015, ηp^2^ = 0.16 (**Table 3**).

**Table 3:** Hierarchical linear regression with K10 total score (as dependent variable).

Predictors	B	SE	*β*	*t*	95%IC Lower Limit	95%IC Upper Limit	p-value	∆F	∆R^2^
Step 1							< .001	5.66	.09
1. Gender (coded as 1 = female, 2 = male)	-4.51	1.15	-.26	-3.94	-6.77	-2.26	< .001		
2. Age (in years)	-.71	.26	-.18	-2.76	-1.21	-.20	.006		
3. Sexual orientation (coded as 1 = heterosexual, 2 = other)	.04	1.41	.00	.03	-2.74	2.81	.980		
4. Perceived income level	-1.47	.99	-.10	-1.48	-3.42	.49	.140		
Step 2							< .001	42.53	.15
5. MSPSS	-.25	.04	-.39	-6.52	-.32	-.17	< .001		
Step 3							.020	5.96	.02
6. L-TFSV	.28	.12	.15	2.44	.05	.51	.015		

Note. SE, Standard Error; CI, Confidence Interval; MSPSS, Multidimensional Scale of Perceived Social Support; L-TFSV, Total Lifetime TFSV victimization; K10; Kessler Psychological Distress Scale.

### 3.5 Moderation analyses

**Figure 1:**
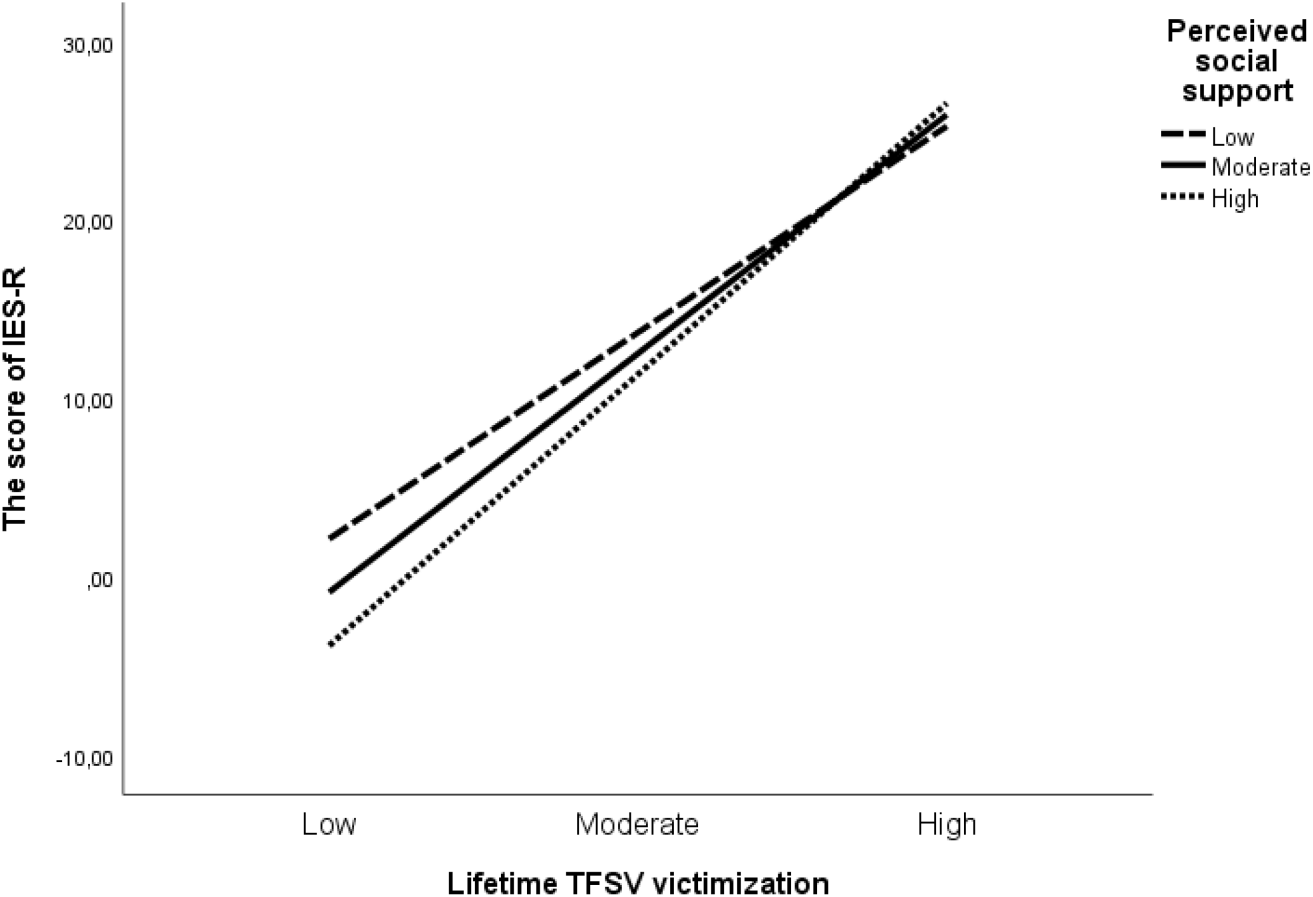
Simple slope analysis of the interaction between lifetime TFSV victimization and perceived social support on the score of IES-R.

For the possible moderator effect of social support (i.e., the scores of MSPSS) on the relation between lifetime TFSV victimization and the IES-R, a moderation analysis using Hayes Macro (model 1) was performed. It was expected that perceived social support would moderate the association between lifetime TFSV victimization and subjective distress related to traumatic experiences. Since in the regression analysis, gender was found to be a significant predictor of the IES-R scores (**Table 2**), in the moderation analysis, gender was entered as a covariate. According to the results, the overall model was significant, *F* (4, 150) = 40.87, *R* ^2^ = 0.52, *p* < 0.001. The main effects of lifetime TFSV victimization [*b* = 2.78, *t* (150) = 11.00, *p* < 0.001, CI[2.2823, 3.2822]] and gender [*b* = -5.24, *t* (150) = -2.04, *p* = 0.043, CI[-10.3108, -0.1638]] were significant but the main effect for perceived social support was not significant, [*b* = -0.09, *t* (150) = -0.99, *p* = 0.324, CI[-0.2621, 0.0872]]. However, the interaction effect (lifetime TFSV victimization X perceived social support) was significant, [b = 0.03, t (150) = 1.99, p = 0.048, CI[0.0003, 0.0562]], indicating that the association between lifetime TFSV victimization and subjective distress related to traumatic experiences changes depending the levels of perceived social support. Slope analysis results showed that lifetime TFSV victimization was a significant predictor of subjective distress related to traumatic experiences at the low [*b* = 2.40, *t* (150) = 8.24, *p* < 0.001)], moderate [*b* = 2.78, *t* (150) = 11.00, *p* < 0.001)], and high [*b* = 3.16, *t* (150) = 4.59, *p* < 0.001)] levels of perceived social support. Contrary to our expectation, as perceived social support increased, the predictive association of lifetime TFSV victimization with the IES-R scores strengthened (see **Figure 1**).

The second moderation analysis was performed to test the possible moderator effect of perceived social support (i.e., the scores of MSPSS) on the relation between lifetime TFSV victimization and psychological distress (i.e., the scores of K10). It was expected that perceived social support would moderate the relation between lifetime TFSV victimization and psychological distress. Since in the regression analysis gender and age were found to be significant predictors of the K10 scores, these variables were entered as covariates in the moderation analysis. According to the results, the overall model was significant, *F* (2, 217) = 15.47, *R* ^2^ = 0.26, *p* < 0.001. Main effects of lifetime TFSV victimization [[*b* = 0.29, *t* (217) = 2.53, *p* = 0.012, CI[0.0629, 0.5093]], perceived social support [*b* = -0.24, *t* (217) = -6.41, *p* < 0.001, CI[-0.3168, -0.1678]], gender [[*b* = -5.11, *t* (217) = -4.66, *p* < 0.001, CI[-7.2762, -2.9494]], and age [[*b* = -0.71, *t* (217) = -3.04, *p* = 0.003, CI[-1.1639, -.2475]] were significant. However, the interaction effect (lifetime TFSV victimization X perceived social support) was not significant, [*b* = 0.01, *t* (217) = 1.43, *p* = 0.155, CI[-0.0036, 0.0229]], indicating that the association between lifetime TFSV victimization and psychological distress did not change depending the levels of perceived social support.

## 4 Discussion

In our study, we firstly conducted a comprehensive psychometric evaluation of the TFSV Victimization Scale through the administration of the Italian translated version to a sample consisting of university students (aged 18-25 years-old) randomly selected from the general population. Overall, our findings support the TFSV-VS as a valid and reliable tool for identifying lifetime and 12-months TFSV victimization in Italian young adults. Indicating that all 21 items of the Italian version are aligned with the general dimension of the original TFSV-VS by Powell and Henry (6). We explored the technology-facilitated/assisted sexual violence phenomenon in our cohort of Italian young adults, by exploring the impact of gender, sexual orientation, age in all four dimensions of the phenomenon as explored through the use of the TFSV-VS (i.e., digital sexual harassment, image-based sexual abuse, sexual aggression/coercition, and gender and/or sexuality-based harassment).

In our sample, around 78.6% of participants dramatically declared to have experienced at least one situation of TFSV victimization in their lifetime, mostly represented by digital sexual harassment (i.e., uninvited behavior that explicitly communicate sexual desires or attention towards another individual) in 70.4% of the sample. Moreover, the second most prevalent TFSV-related situation was gender and/or sexuality-based harassment (i.e., unwelcome comments that insult or cause discomfort to another individual on the basis of a person’s gender, sexuality or sexual orientation), reported in around 43% of the sample. These findings are in line with those already documented in previous Australian and Canadian studies recruiting an adult sample (not selecting only young adults) (6, 16). Powell and Henry (6) found a TFSV prevalence of around 62.3% of the sample, specifically rising up to 71.8% in the 18-24 age group (6). While Canadian study by Snaychuk and O’Neill (16) reported a slightly higher percentage of around 84.3% in university students.

Interestingly, a gender-based effect has been found in our sample, being females those who are more likely to be exposed to lifetime TFSV victimization situation/s than males, as already documented in previous studies which reported approximately a 3.5-fold increase in experiencing TFSV among females compared to the male counterpart (6, 27, 28). Moreover, our findings clearly indicated that females were more likely to be particularly exposed to lifetime digital sexual harassment and gender and/or sexuality-based harassment victimization, in comparison with their male counterpart. Moreover, participants of our study who declared to belong to sexuality and gender minority (SGM) were more likely to be exposed to digital sexual harassment, gender and/or sexuality-based harassment victimization, and the combined lifetime TFSV victimization in their lifetime. Furthermore, around 56.1% of participants declared to have experienced at least one episode of TFSV-related victimization in the last 12 months, mainly represented by digital sexual harassment in 46.6% and gender and/or sexuality-based harassment in 29.6% of the sample. These findings did not appear to be influenced by gender, except for gender and/or sexuality-based harassment victimization in the last 12 months, which appeared to be most likely represented among females, compared to males. Moreover, participants with sexual orientations other than heterosexual ones were more likely to be exposed to gender and/or sexuality-based harassment victimization in the last 12 months. Previous studies already documented the role of gender (particularly younger females) and sexual orientation as predictors of a higher risk to be victims of TFSV (29, 30), by suggesting that TFSV is “fundamentally an issue of gender” (18).

Furthermore, it has been well documented and described in the previous literature the negative impact of exposure to sexual violence on individual’s mental health, being more likely associated with the occurrence of subjective psychological and posttraumatic distress, including the onset of clinically relevant depression, anxiety, somatization symptomatology as well as the occurrence of an adjustment disorder or a posttraumatic stress disorder (PTSD) (31–33). Similarly, victimization following TFSV was found to be associated with detrimental mental health outcomes, including depression, anxiety, PTSD, suicidal ideation, substance use issues and negative interpersonal impacts (28, 34–36). Therefore, in our study we evaluated the impact of technology-assisted/facilitated sexual violence on individual’s mental health, by specifically investigating its role in a cohort of Italian university students, by exploring the association between the psychological distress (by using K10) and subjective distress related to traumatic experiences (by using IES-R) with the TFSV-VS, as well as the potential other socio-demographic predictive variables influencing this association. According to the hierarchical regression analysis models, when we evaluated the predictive association of lifetime TFSV victimization with subjective distress related to traumatic experiences (IES-R), we found that only being female was significantly associated with higher subjective distress related to technology-assisted traumatic experiences, particularly lifetime TFSV victimization. Another hierarchical regression analysis also found that being female and younger are significantly and positively associated with the experience of being exposed to higher psychological distress following a lifetime TFSV victimization experience, as already documented in previous studies (18, 29, 37).

Finally, as previous literature supported the hypothesis of a protective effect of perceived social support in the association between the experience of victimization of a technology-facilitated sexual violence and the emergence of a psychological distress related to TFSV Victimization experience (16), we investigated the moderator role of the perceived social support (as assessed by using the MSPSS). According to two hierarchical regression models in which both the IES-R and K10 were considered as dependent variables, it was found that the levels of perceived social support negatively predicted the levels of distress in our sample, by suggesting a possible protective role of higher levels of social support in the emergence of a psychological distress related to be victim of a technology-assisted sexual violence on the Internet. Indeed, despite the moderation analysis confirmed that the association between lifetime TFSV victimization and subjective distress related to traumatic experiences (as measured by IES-R) depend on the levels of perceived social support, contrarily to our initial research hypothesis, the predictive association of lifetime TFSV victimization with the IES-R scores strengthened with the increase of the perceived social support. These findings indeed suggested that the level of perceived social support does not seem to weaken the relationship between TFSV and the occurrence of TFSV-related psychological distress. Moreover, according to our findings, the association between lifetime TFSV victimization and psychological distress (as assessed by K10) did not change depending on the levels of perceived social support, which did not seem to act as moderator between these two variables. Consequently, considering these findings, one could argue that probably the technology-mediated psychopathological trajectory could be different from that experienced in an in-person sexual violence. However, further studies should confirm these findings, by comparing a sample of subjects who experienced sexual violence only in the real life versus a sample who experienced only TFSV, in order to compare if any differences and/or similarities could exist depending on the level of perceived social support.

Overall, our findings may significantly contribute to the knowledge and understanding of the extent and nature of the TFSV Victimization phenomenon in Italian youths. In fact, at the time of the present writing, there are no studies and/or research published investigating the gendered extent and nature of TFSV in the Italian context. The choice to target the study only to young adults also was supported by the hypothesis that the technology-assisted phenomena represent nowadays an age-specific critical and urgent need to be investigated within the youth generation, much more likely exposed to Internet and more frequently users of technological devices and contents on the web (38). Interestingly, our findings confirmed results coming from the original validation study of TFSV-VS carried out on Australians, which clearly reported a gendered trend of TFSV as well as a phenomenon also influenced by belonging to the SGM community.

However, our findings should be discussed and generalized also considering the following set of potential limitations. Firstly, the cross-sectional study design limits to achieve a deeper understanding of the causal relationship between the potential impact of TFSV on victims’ mental health, quality of life and perceived psychological distress associated or not with the subjective technology-assisted sexual traumatic experience. Therefore, further longitudinal studies should be carried out to replicate our findings, by using the TFSV-VS, IES-R, K10 and other assessment tools more specific to investigate anxiety, depression and stress symptomatology with periodic follow-ups in order to assess the clinical course of mental health issues depending on the frequency and type of TFSV exposure, other individuals’ socio-demographic and personality features. Secondly, although the online survey may help clinicians in collecting data in a less perceived stigmatizing and friendly setting, especially for those subjects less prone to seek help from mental health professionals, the online recruitment strategy could not be always representative of the entire sample of Italian young adults. Moreover, despite the choice to address the survey only to young adults was *a priori* established by the research team, due to epidemiological reasons, further studies should also explore the TFSV phenomenon across all ages, to confirm whether this gendered trend could be influenced by the age or other social variables, not specifically investigated in our study (such as the participants’ educational level, the number of previous experiences of sexual, emotional and/or physical traumatization and/or abuse, previous childhood trauma experiences, family context in terms of attachment styles, coping strategies, and so forth). Thirdly, even though in our study we did not confirm the protective role of perceived social support in moderating the relationship between the experience of TFSV and the occurrence of psychological distress, it is also true that we could hypothesize that technology-mediated phenomenon could act in following a different psychopathological trajectory compared to in-person sexual violence. Indeed, further studies should be carried out to clearly confirm our findings and investigate as well as characterize the phenomenon depending on the experienced context.

In conclusion, our findings could be useful for policy, prevention and treatment future directions as well as to inspire future research studies investigating TFSV phenomenon in a more deeper manner. Our findings could be evaluated and potentially integrated in implementing a country-based national policy response able to increase preventive strategies specifically targeting TFSV and technology-mediated forms of discrimination, sexuality and/or gender-based discrimination, inequality and victimization. Our study also demonstrated that gender is not the only predictor of TFSV, by clearly underlining that also the highest prevalence of TFSV experiences are also manifested by younger population, as well as SGM communities. Therefore, these populations should represent the first-line target population for policy and preventive initiatives, also in the Italian context. Furthermore, there is also the urgent need to provide not only formal preventive strategies, but also supporting responses able to target TFSV among those most vulnerable populations (females, youngsters and SGM communities), with the aim to take care about their specific tailored needs and differentiating target interventions depending on the different TFSV victims, survivors and perpetrators. A further clinical implication to be addressed regards the need to implement gender- and sex-specific therapeutic interventions to TFSV victims who necessarily require more accurate and adequate settings and interventions.

## Data Availability

The raw data supporting the conclusions of this article will be made available by the authors, without undue reservation.
